# Secondary pulmonary conventional chordoma arising from primary sarcomatoid chordoma of the sacrum: A case report

**DOI:** 10.3892/ol.2014.2100

**Published:** 2014-04-28

**Authors:** JIA-HONG CHEN, KUAN-YU CHEN, DUENG-YUAN HUENG, JONG-SHIAW JIN

**Affiliations:** 1 Department of Medicine, Division of Hematology/Oncology, Tri-Service General Hospital, National Defense Medical Center, Taipei 114, Taiwan, R.O.C.; 2 Hualien Armed Forces General Hospital, Hualien 920, Taiwan, R.O.C.; 3 Department of Neurological Surgery, Tri-Service General Hospital, National Defense Medical Center, Taipei 114, Taiwan, R.O.C.; 4 Department of Pathology, Tri-Service General Hospital, National Defense Medical Center, Taipei 114, Taiwan, R.O.C.

**Keywords:** chordoma, sarcomatoid, conventional, transformation

## Abstract

Chordomas are low- to intermediate-grade malignant tumors that recapitulate the notochord. Chordomas belong to the dysontogenetic bone tumors and appear primarily in the region of the axial skeleton. Chordomas are divided into conventional, chondroid, sarcomatoid and dedifferentiated subtypes. The different subtypes of chordoma have varied survival periods. According to the literature to date, secondary pulmonary and lymph-node metastases occur most frequently, followed by liver, bone and skin metastases. To the best of our knowledge, there has been no previous report of one subtype of chordoma metastasizing or transforming into another subtype with a different histopathology. This study presents a 24-year-old man with secondary pulmonary conventional chordoma arising from a primary sarcomatoid chordoma of the sacrum. The patient was alive at the end of November, 2009 and the survival time exceeded eight years. This is the first case of a patient with primary sarcomatoid chordoma of the sacrum with complete remission in whom a secondary pulmonary conventional chordoma arose from the primary cancer.

## Introduction

Chordomas are low- to intermediate-grade malignant tumors that recapitulate the notochord. Etiologically, chordomas belong to the dysontogenetic bone tumors and occur predominantly in the region of the clivus (1). Corresponding to their course of embryological development, chordomas primarily appear in the region of the axial skeleton and typically occur in midlife, from the ages of 40 to 60 years, and primarily affect men (2).

The histological appearance of classical chordoma is of a lobulated tumor composed of groups of cells separated by fibrous septa. The cells have small round nuclei and abundant vacuolated cytoplasm, occasionally described as physaliferous (having bubbles or vacuoles) (3).

Although chordomas are locally invasive, metastasis has been reported in 10–42% of cases. However, these may be the result of the misdiagnosis of mucus-producing carcinomas of the rectum as chordomas in certain case studies (4–6). Previous studies suggest that secondary pulmonary and lymph-node metastases occur most frequently, followed by liver, bone and skin metastases (4,7).

According to the current literature, no case of a chordoma subtype that has metastasized or transformed into another subtype with a different histopathology and immunoreactivity has been previously reported. This study presents a case of secondary pulmonary conventional chordoma arising from a primary sarcomatoid chordoma of the sacrum.

## Case report

**Figure 1:**
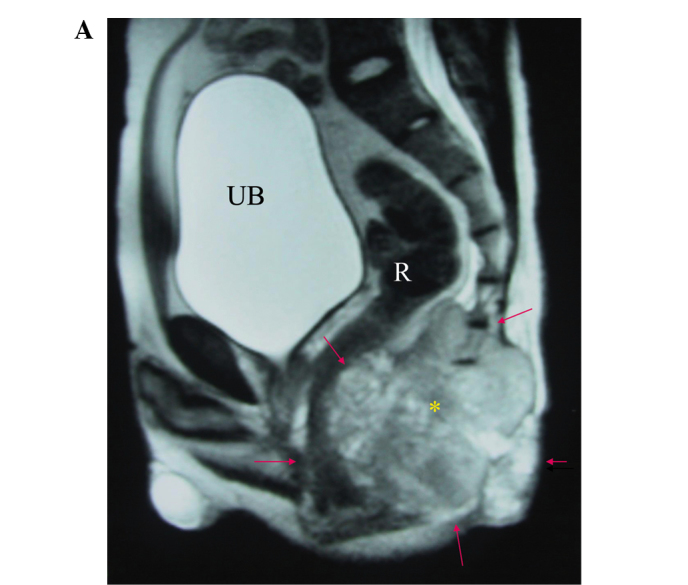

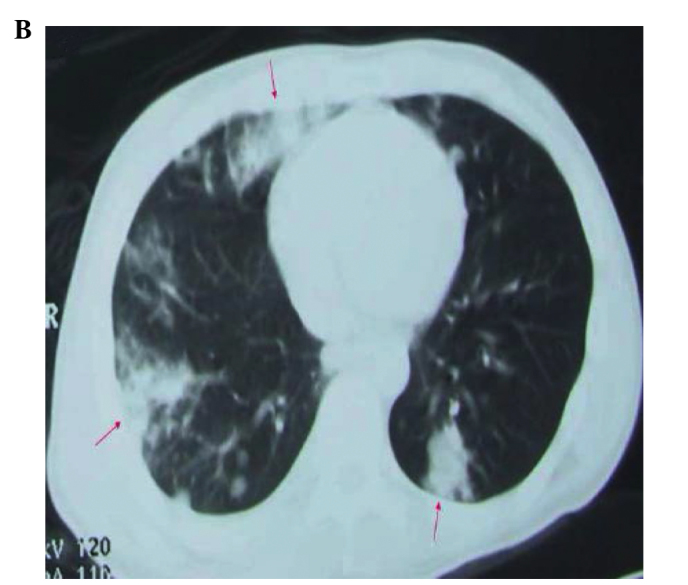
(A) Gadolinium contrast-enhanced magnetic resonance imaging shows a large tumor at the sacrum, with compression of the intestine and urinary bladder. The pink arrows indicate the tumor edge and the asterisk indicates the center of the tumor. (B) Multiple metastases in the bilateral lung fields. The pink arrows indicate the the tumor edge.

**Figure 2:**
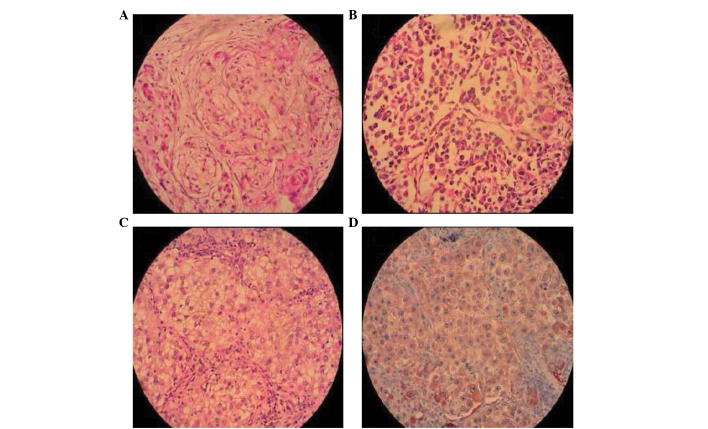
Two different subtypes of chordoma identified in one patient. (A) Histopathology of the conventional chordoma of the sacrum revealed a lobular architecture, eosinophilic vacuolated (physaliphorous) cytoplasm and a mucoid matrix (H&E stain). (B) Histopathology of the sarcomatoid chordoma of the sacrum revealed a lobular architecture with spindle tumor cells, eosinophilic vacuolated (physaliphorous) cytoplasm and a mucoid matrix (H&E stain). (C) Histopathology of the conventional chordoma of the lung revealed a lobular architecture, eosinophilic vacuolated (physaliphorous) cytoplasm and a mucoid matrix (H&E stain). (D) Immunohistochemistry of the conventional chordoma of the lung was positive for cytokeratin (cytokeratin stain). Original magnification, ×100. H&E, hematoxylin and eosin.

A 24-year-old man was referred to the Tri-Service General Hospital (Taipei, Taiwan, R.O.C) presenting with a palpable sacral mass and constipation of two months in duration. A magnetic resonance imaging (MRI) examination of the sacral spine revealed a large mass (measuring 13×8×7 cm) with compression of the colon (Fig. 1A). Based on the clinical and radiological characteristics, the patient underwent an exploratory laparotomy with debulking of the tumor. The gross findings of the sacral tumor included multilobulated, soft, gelatinous masses with deceptively discrete margins. Microscopic analysis revealed a lobular architecture, spindle tumor cells with eosinophilic vacuolated cytoplasm and a mucoid matrix. The pathological diagnosis was a sarcomatoid-type chordoma. Immunohistochemical staining of the sacral tumor was positive for cytokeratin (CK), epithelial membrane antigen (EMA), vimentin, S100 and periodic acid-Schiff (Fig 2A and B). Postoperative adjuvant radiotherapy (50 Gy) was performed.

After one year, the patient presented to the Tri-Service General Hospital with dizziness and unstable gait of one week in duration. Computed tomography (CT) of the brain revealed a mass lesion over the right cerebellar vermis. A suboccipital craniotomy was performed, with removal of the mass and the placement of a ventriculoperitoneal shunt. The pathological findings identified a sarcomatoid-type metastatic chordoma. Immunohistochemical staining was positive for CK, EMA, vimentin and S100. Intracranial whole-brain radiotherapy and adjuvant chemotherapy with methotrexate were administered to the patient.

After two years, a CT examination of the lungs was performed due to a chronic cough, revealing multiple lesions, enlarged lymph nodes and suspected metastases over the right lower lobe (Fig. 1B). A thoracotomy with a wedge resection of the right lower lobe was performed. The final histopathological evaluation of the lung tumor tissue following an immunohistochemical examination revealed a metastatic conventional chordoma (Fig. 2C and D). Cisplatin-based chemotherapy was administered and the disease was stable after four courses of treatment. However, disease progression was noted after six months and persistent salvage chemotherapy was administered. The patient remained alive at the end of November, 2009 and, thus, the survival time had exceeded eight years.

## Discussion

In the present case, the patient suffered from two different subtypes of chordoma. The first was a sarcomatoid chordoma that responded completely to an exploratory laparotomy with debulking of the tumor and postoperative radiotherapy, with no evidence of residual disease. The secondary chordoma was the conventional subtype. To the best of our knowledge, this study is the first to present the case of a different subtype of chordoma arising at a metastatic site.

The majority of patients with conventional-type chordoma are 50–70 years old and 50% of these tumors arise in the sacrococcygeal region (8). The pathological findings of conventional chordoma include sheet- or cord-like tumor cells floating in the myxoid stroma, with abundant vacuolated cytoplasm (physaliphorous cells). The mean survival is 4.1 years. In sarcomatoid chordomas, the tumor cells display a storiform architecture with large, pleomorphic nuclei. A transitional characteristic distinguishing sarcomatoid chordoma from conventional chordoma is positive CK immunoreactivity in the sarcomatoid component, which is required for a pathological diagnosis (9–12). Prognosis of chordoma is associated with the extent of surgical removal; a five-year survival of 35% has been reported with incomplete resection if followed by conventional radiation therapy (13).

In the dedifferentiated type of chordoma, the pathological findings show a sharp demarcation between the conventional chordoma and the high-grade sarcoma, with no transitional characteristics between the two components, and negative CK immunoreactivity in the sarcoma component (14–16).

The primary subtype of the chordoma affects the survival period and the progressive characteristics of the tumor, including local relapses, surgical pathway seeding and progressive distal metastasis (5–43% of patients develop metastatic tumors in the skin, subcutaneous tissue, bones, lungs and lymph nodes) (17). Therefore, the subtype of a metastatic lesion correlates with its histological differentiation and the duration of the clinical course.

Standard treatments for conventional chordoma are maximal surgical resection and subsequent radiotherapy (50 Gy), even proton-beam therapy. These treatments may improve morbidity and mortality (18). However, a two-year mean overall survival period has previously been reported for hematogenous metastases that include a sarcomatous component (19). The patient in the present case was alive at the end of 2009, despite disease progression with metastases. In summary, to the best of our knowledge, this study presents the first patient with primary sarcomatoid chordoma of the sacrum with complete remission, in whom a secondary pulmonary conventional chordoma arose from the primary tumor.
